# Factors predicting team climate, and its relationship with quality of care in general practice

**DOI:** 10.1186/1472-6963-9-138

**Published:** 2009-08-04

**Authors:** Teik T Goh, Martin P Eccles, Nick Steen

**Affiliations:** 1 Institute of Health & Society, Newcastle University, 21 Claremont Place, Newcastle upon Tyne, NE2 4AA, UK; 2 Northumbria General Practice Vocational Training Scheme, Northern Deanery, 10-12 Framlington Place, Newcastle upon Tyne, NE2 4AB, UK

## Abstract

**Background:**

Quality of care in general practice may be affected by the team climate perceived by its health and non-health professionals. Better team working is thought to lead to higher effectiveness and quality of care. However, there is limited evidence available on what affects team functioning and its relationship with quality of care in general practice. This study aimed to explore individual and practice factors that were associated with team climate, and to explore the relationship between team climate and quality of care.

**Methods:**

Cross sectional survey of a convenience sample of 14 general practices and their staff in South Tyneside in the northeast of England. Team climate was measured using the short version of Team Climate Inventory (TCI) questionnaire. Practice characteristics were collected during a structured interview with practice managers. Quality was measured using the practice Quality and Outcome Framework (QOF) scores.

**Results:**

General Practitioners (GP) had a higher team climate scores compared to other professionals. Individual's gender and tenure, and number of GPs in the practice were significantly predictors of a higher team climate. There was no significant correlation between mean practice team climate scores (or subscales) with QOF scores.

**Conclusion:**

The absence of a relationship between a measure of team climate and quality of care in this exploratory study may be due to a number of methodological problems. Further research is required to explore how to best measure team functioning and its relationship with quality of care.

## Background

Quality of care in general practice is of interest to decision makers and the general public [1-4]. Ferlie and Shortell suggest that care provision and its improvement could be considered at four levels: system, organisation, team and individuals [5]. As much of the clinical care delivered in primary care in the UK is provided by the general practice team [6-8], the question of whether or not how such teams function and their approach to collaborative working influences the quality of care that patients receive is of interest [9-11].

Two decades ago, Bond et al (1985) identified a lack of multidisciplinary collaboration in primary care teams [12]. Subsequent studies have suggested that primary care team have significantly lower team scores compared to teams in other services and industries [13,14]. Various factors were thought to act as facilitators or barriers to effective team work as reported in a recent literature review [15]. Its thematic analysis of literature suggests that premises, team size and composition, organisational support, team meetings, clear goals and objectives, and audit were related to interprofessional team working in primary care [15]. Barriers to effective team work can be viewed at different levels including the organisation and the practice levels. In primary care there are a wide range of team members who may have their own goals and priorities. They come from disciplines that have differing philosophies [16-18]. Thus, professional sub-groups may have distinct views about the team and team working.

The theoretical framework for studying team processes is predominated by the Input-Mediator-Outcome (IMO) model which suggests complex, dynamic and nonlinear states with cyclical feedbacks [9,19,20]. The *Inputs *describe the organisational context within which the team operates, and includes the team context and the characteristics of its individual professionals; the skill-mix of the teams. *Mediators *are the interactions of the team and its environment emerging states and *Outcomes *are the end result of the team's action, the performance and quality of the team outcome that can be assessed through various criteria [19,21].

In the UK, general practices have received performance-linked reimbursement under the Quality and Outcomes Framework since 2004. The revised 2006/07 framework is comprised of indicators of: clinical care across 19 conditions; practice organisation; patient experience; additional services; and holistic care. Within each of these areas points are scored for achieving specified indicators. With a total of 1000 points, 655 are awarded to clinical care and 345 to all of the other areas [22]. The process of data collection also allows the calculation of practice specific disease prevalence. Previous work has examined the relationship between general practice characteristics and quality of care as measured by the Quality and Outcome Framework (QOF) [23]. Social deprivation was found to be an independent predictor for lower quality of primary care, whilst being a training or a larger group practices predict higher quality [23].

### Team climate

Team climate can be measured by using the Team Climate Inventory (TCI), was developed by Anderson and West for measuring innovation in teams [24,25]. The TCI has been validated and applied in a variety of settings, including primary and secondary care [24-28]. It has four sub-scales: (1) 'Vision' which represents the team members' perceived clarity, sharedness and attainability of the team's objectives; (2) 'Participative safety' as members' psychological safety and participation in information sharing and decision making; (3) 'Task orientation' as members' reflection on appraisal, feedback and performance monitoring of work; and (4) 'Support for innovation' measures the perceived help in applying of new ideas and improvement [24].

Anderson and West used the criteria 'proximal work group' in the analysis of shared perception of team climate. The work group can be the 'permanent or semi-permanent team to which individuals are assigned, whom they identify with, and whom they interact with regularly in order to perform work-related task' [24]. Effectively this shared perception prompts the individuals to interact, to work collectively towards particular shared goals, and to develop to some extent a common understanding and norms of practice [24].

A systematic review of literature on the use of the TCI in UK primary care settings showed that previous studies on team climate and effectiveness used teams nominated through a local team organising team building workshops and used self-reported effectiveness (Goh T, Eccles M: Team climate and quality of care in primary health care: a review of studies using Team Climate Inventory in the United Kingdom, Submitted) [29]. Two other studies used general practice based teams and suggested that higher team climate scores were significantly associated with better management of certain chronic conditions and higher patient self-reported satisfaction [30,31]. However this was not repeated in a subsequent study by the same group [32].

### Objective

This study aimed to identify individual and practice level correlates of team climate as measured by the Team Climate Inventory (TCI), and to explore the relationship between the TCI and quality of care as measured by QOF scores.

## Methods

### Setting and participants

The study took place between May and July 2008. All 29 general practices serving the South Tyneside Primary Care Trust (PCT) in the northeast of England were approached to participate. These practices provide a full range of primary care services, each to a registered population (approx 1700 patients per full time general practitioner). They are financially contracted to the PCT and they perform a gatekeeper function in terms of access to secondary care.

Practice level consent was obtained to approach individuals, and all staff employed or attached to the practice were invited to participate in the study. The types of staff included were general practitioners (GP), practice nurses (PN), administrative staff (including practice managers, secretaries, and receptionists), community nurses (including district nurses, health visitors), and other professionals (including counsellors, midwives, practice pharmacists, physiotherapists, podiatrist etc). Temporary or trainee staff such as GP registrars and trainee receptionists was excluded.

### Data collection

The study questionnaire included 14 questions (Table 1) modified from the short version of TCI developed by Kivimaki and Elovainio [26,33], plus four demographic questions (gender, year of birth, year joining the practice, sessions worked per week). Responses to the TCI items were on a 5-point Likert scale (strongly disagree to strongly agree). Data on the characteristics of practices were collected using brief structured interview with practice managers. The most recent available QOF and disease prevalence data (2006/07) was obtained from the NHS Information Centre [22]. The questionnaire was distributed to all the staff via the practice manager. Non-respondents were sent a reminder letter and a further copy of the questionnaire at two weeks and four weeks after the initial distribution.

**Table 1:** Items on the TCI short version (modified from Kivimaki et al 2007 [42])

Questions*		Subscale
1	How far are you in agreement with the objectives of your practice?	Vision
2	To what extent do you think the objectives of your practice are clearly understood by other members of the practice?	
3.	To what extent do you think the objectives of your practice can actually be achieved?	
4.	How worthwhile do you think these objectives are to the practice?	

5.	We have a "we are in it together" attitude	Participatory safety
6.	People keep each other informed about work related issues in the practice	
7.	People feel understood and accepted by each other	
8.	There are real attempts to share information throughout the practice	

9.	Are members of your practice prepared to question the basis of what the practice is doing?	Task orientation
10.	Does the practice critically appraise potential weaknesses in what it is doing to achieve the best possible outcome?	
11.	Do members of the practice build on each other's ideas to achieve the best possible outcome?	

12.	People in this practice are always searching for fresh, new ways of looking at problems.	Support for innovation
13.	In this practice we take the time needed to develop new ideas.	
14.	People in the practice cooperate to help develop and apply new ideas.	

*We used "practice" throughout the questionnaire to indicate the "work unit" originally stated on Kivimaki et al 2007.

### Data analysis

In order to assess whether levels of agreement within practices were acceptable, for each practice, for each subscale of the TCI, we calculated a measure of inter-rater agreement (r_wg(j)_) [34,35] assuming a uniform null distribution, using SPSS [36]. The QOF scores of participating and non-participating practices were compared using independent sample t-tests.

The association between respondents' perceptions of team climate and indicators of good quality of care was assessed using Pearson product moment correlation coefficients; the mean TCI scores (both total scores and sub-domain scores) for each practice were correlated with the practice QOF scores.

Regression analysis was used to identify individual and practice level factors that were associated with the respondents' perception of team climate. This was done by using the 'xtreg' procedure in Stata [37] to fit a mixed effects model; the respondent's TCI score was included as the dependent variable, the individual or practice level characteristic was included as a fixed effect and variation between practices and variation between respondents within practices were modelled as random effects. Factors that were significant (alpha level 0.05) were then included in a multiple regression analysis to explore their relative contribution in explaining the observed variation in TCI scores. This was done using the mixed effects models approach described above. Initially all identified factors were included simultaneously as fixed effects. The factors were then excluded one at a time to assess the importance of that factor given the inclusion of all the other factors in the model; this was achieved by comparing changes in -2 log likelihood with the percentage points of the appropriate chi-squared distribution.

### Ethics approval

The study received ethics approval from the Newcastle and North Tyneside 2 Research Ethics Committee (08/H0907/35).

## Results

### Description of general practices and non-participatory analysis

Fourteen of the 29 practices approached agreed to participate in the study. A total of 283 health and non-health professionals within these 14 practices participated in the study. Response rates and demographic characteristics of the respondents are shown in Table 2. While subjects with any missing data were excluded from subsequent analyses, as the mean (range) percentage practice response rate to the questionnaire was 78.6% (42.9 – 94.1) all practices were included in the analysis.

**Table 2:** Practice staff approached: response rates and demographic data.

	Number approached	Number responded	Response rate (%)	Practice response rate (% (range))	Number analysed	Mean age* (years)	Mean tenure* (years)	Mean session/week*	Gender*	
									
									Female	Male
**GP**	61	50	82.0	80.8 (25.0–100)	49	43.2	11.6	6.55	21	28
**Practice Nurse**	43	35	81.4	79.7 (0–100)	33	50.1	8.1	6.18	31	2
**Admin**	132	119	90.2	89.4 (62.5–100)	109	46.0	9.0	6.87	108	1
**Community Nurse**	63	44	69.8	68.2 (0–100)	25	48.1	9.6	4.84	25	0
**Others**	49	35	71.3	63.3 (0–100)	33	41.8	5.0	1.91	24	9
**Total**	350	283	80.6	78.6 (42.9–94.1)	249	45.7	8.9	5.85	209	40

Admin = Practice managers and receptionists; Community nurses = district nurses and health visitors; Others = Counsellors, midwives, practice pharmacists and physiotherapists. *Mean for number of participants analysed (n = 249)

Amongst the 249 participants included in the analysis, 140 were health professionals including 49 GPs (21 female and 28 male GPs), and 109 were non-health professionals including 14 practice managers.

The characteristics of participating practices, their practice mean TCI scores, QOF scores and disease prevalences are shown in Table 3. There were no significant differences in terms of list size and QOF data between the 14 participating and 15 non-participating practices.

**Table 3:** Characteristics of participating and non-participating practices.

Variable	Participating practice (N = 14)		Non-participating practice (N = 15)		P value
		
	Mean	(95% CI)	Mean	(95% CI)	
**Practice profile**					
Practice list size	5310	(3500, 7121))	5407	(3649, 7165)	0.63
GP appointments/week	456	(419, 493)	-	-	-
Total number of staff	28.3	(27.1, 29.4)	-	-	-
General practitioners	5.3	(4.9, 5.7)	-	-	-
Practice nurse	3.4	(3.3, 3.6)	-	-	-
Administrative staff	11.0	(10.4, 11.5)	-	-	-
Community nurse	5.0	(4.7, 5.3)	-	-	-
Other professionals	3.6	(3.4, 3.9)	-	-	-
Number of Partners	3.0	(2.7, 3.2)	-	-	-
Partner's sessions/week	6.3	(5.9, 6.7)	-	-	-
Number of Salaried GPs	2.3	(2.1, 2.5)	-	-	-
Salaried GP sessions/week	5.6	(5.3, 6.0)	-	-	-
PN sessions/week	7.2	(7.0, 7.4)	-	-	-
**Team climate**					
Total TCI scores	50.40	(48.02, 52.57)	-	-	-
Vision	14.15	(13.78, 14.53)	-	-	-
Participatory safety	15.17	(14.77, 15.57)	-	-	-
Task orientation	10.05	(9.77, 10.33)	-	-	-
Support for innovation	11.03	(10.76, 11.30)	-	-	-
**QOF scores**					
Total QOF	958.27	(936.45, 980.10)	964.51	(941.54, 987.47)	0.66
Clinical domains	624.20	(605.73, 642.67)	634.23	(616.44, 652.02)	0.80
Organisational domains	172.75	(169.32, 176.18)	172.04	(170.50, 175.37)	0.54
Holistic care	17.52	(15.45, 19.59)	17.99	(17.72, 20.25)	0.63
**Disease prevalence**					
CHD (%)	5.24	(4.89, 5.60)	4.81	(4.32, 5.30)	0.07
Stroke (%)	2.34	(2.13, 2.55)	2.10	(1.85, 2.34)	0.06
Hypertension (%)	15.93	(14.21, 17.65)	14.23	(12.52, 15.94)	0.07
Diabetes mellitus (%)	4.40	(4.14, 4.67)	4.18	(3.87, 4.50)	0.13
COPD (%)	3.03	(2.33, 3.74)	2.81	(2.31, 3.31)	0.29
Asthma (%)	5.53	(4.93, 6.13)	5.24	(4.78, 5.71)	0.21
Heart failure (%)	2.73	(1.94, 3.52)	2.77	(1.76, 3.78)	0.53

CHD = Coronary Heart Disease; COPD = Chronic Obstructive Pulmonary Disease

### Team climate and individual and practice characteristics

The within practice inter-rater agreement r_wg(j) _varied from 0.82 to 0.88 with 86% being above the recommended threshold of 0.7 (Table 4). Intra-class correlation coefficients were in the same order as reported by Anderson and West. [24] Table 5 shows the correlations between the subscales and total scores of the TCI. The mean overall and subgroup scores for TCI and its subscales by professional group are shown in Table 6.

**Table 4:** Inter-rater agreement (r_wg(j_, IRA) and intra-class correlation coefficient.

Items	Mean IRA	Range	% >0.7*	**ICC**†	95% CI
**All TCI items**	0.96	0.90–0.98	100.0	0.27	0.15, 0.34
**Vision**	0.88	0.73–0.97	100.0	0.19	0.06, 0.28
**Participatory safety**	0.82	0.52–0.96	92.9	0.24	0.12, 0.32
**Task orientation**	0.83	0.58–0.93	85.7	0.27	0.16, 0.35
**Support for innovation**	0.86	0.66–0.98	92.9	0.23	0.11, 0.31

*Percentage of practices above 0.7 threshold

† ICC (Intra-class correlation coefficient) is for the baseline model

**Table 5:** Correlation between mean practice TCI scores and QOF scores (N = 14).

Practice mean scores	TCI	Vision	Participatory Safety	Task Orientation	Support for Innovation
**Total TCI scores**	1				
Vision	0.8993*	1			
Participatory Safety	0.9463*	0.7744*	1		
Task Orientation	0.9803*	0.8522*	0.9220*	1	
Support for Innovation	0.9008*	0.7245*	0.7958*	0.8835*	1
**Practice mean total QOF score**	-0.1522	-0.2265	-0.2285	-0.0953	0.0382
Clinical Domain	-0.1067	-0.2165	-0.1888	-0.0357	0.1050
Organisational Domain	-0.2143	-0.0309	-0.2458	-0.2693	-0.2595

* p < 0.05

**Table 6:** Mean scores for total TCI and subscales by professional subgroups.

Profession	Team climate (95% CI)		Vision (95% CI)		Participatory Safety (95% CI)		Task Orientation (95% CI)		Support for Innovation (95% CI)	
**Total (n = 249)**	50.40	(49.27, 51.52)	14.15	(13.78, 14.53)	15.17	(14.77, 15.57)	10.05	(9.77, 10.33)	11.03	(10.76, 11.30)

**General Practitioners (n = 49)**	55.35	(53.21, 57.48)	15.37	(14.70, 16.03)	16.96	(16.20, 17.72)	11.08	(10.48, 11.68)	11.94	(11.48, 12.39)

**Practice nurses (n = 33)**	47.70	(44.04, 51.35)	13.48	(12.15, 14.81)	13.94	(12.51, 15.37)	9.76	(8.92, 10.60)	10.52	(9.71, 11.32)

**Admin staff (n = 109)**	48.90	(47.25, 50.54)	13.79	(13.24, 14.34)	14.55	(13.99, 15.11)	9.70	(9.29, 10.11)	10.86	(10.45, 11.27)

**Community nurses (n = 25)**	48.48	(45.23, 51.73)	13.72	(12.58, 14.86)	15.08	(13.98, 16.18)	9.24	(8.37, 10.11)	10.44	(9.36, 11.52)

**Others (n = 32)**	52.47	(48.91, 55.39)	14.55	(13.38, 15.71)	15.85	(14.67, 17.02)	10.58	(9.85, 11.30)	11.18	(10.44, 11.92)

Administrative staff = practice managers and receptionists; Community Nurses = district nurses and health visitors; Others = counsellors, midwives, practice pharmacists and physiotherapists.

The results of the initial regression analysis are reported in Table 7. The following factors were associated with TCI score: gender (male respondents tended to report higher TCI scores than female respondents); tenure (longer tenure was associated with a higher TCI score); professional role (GPs tended to report higher TCI scores than other respondents); list size (practices with a list size of between 3501 and 7000 reported lower TCI scores than other practices); and number of GPs in the practice (practices with 3 to 5 GPs reported lower TCI scores than other practices).

**Table 7:** Regression analysis: effect of each predictor on TCI score (n = 249)

Model	Fixed components				Random components			-2 log-likelihood	**P**†
			
	Parameter‡	Estimate	95% CI	P	Parameter	σ	95% CI		
**Predictors measured at the level of the individual**									

Baseline	Constant	50.32	48.36, 52.28	0.00	Practices	3.08	1.38, 4.78	1788.02	
					Individuals	8.49	7.72, 9.26		

Age (years)	Constant	47.72	42.46, 52.98	0.00	Practices	3.07	1.38, 4.77	1786.94	0.30
	Age	0.06	-0.05, 0.16	0.30	Individuals	8.47	7.70, 9.24		

Gender*	Constant	49.25	47.43, 51.07	0.00	Practices	2.67	1.09, 4.24	1767.71	<0.001
	Male	6.62	3.79, 9.46	0.00	Individuals	8.18	7.44, 8.92		

Tenure* (years)	Constant	48.88	46.57, 51.20	0.00	Practices	3.19	1.48, 4.91	1782.28	0.02
	Tenure	0.16	0.03, 0.29	0.02	Individuals	8.37	7.62, 9.13		

Sessions (per week)	Constant	48.47	45.43, 51.50	0.00	Practices	3.20	1.47, 4.93	1785.49	0.11
	Session	0.33	-0.07, 0.73	0.11	Individuals	8.43	7.67, 9.19		

Professional role*	Constant	55.04	52.24, 57.84	0.00	Practices	2.88	1.24, 4.51	1764.96	<0.001
	Nurse	-7.66	-11.27, -4.05	0.00	Individuals	8.11	7.38, 8.85		
	Admin	-5.92	-8.70, -3.15	0.00					
	Community nurse	-5.90	-9.89, -1.90	0.00					
	Other	-3.33	-7.02, -0.36	0.08					

**Predictors measured at the level of the practice**									

List size*	Constant (≤ 3500)	52.45	49.73, 55.17	0.00	Practices	2.30	0.77, 3.83	1782.57	0.02
	3501–7000	-4.75	-8.57, -0.93	0.02	Individuals	8.48	7.72, 9.25		
	>7001	-1.35	-5.52, 2.82	0.53					

Number of General Practitioners*	Constant (1 or 2)	53.97	49.76, 58.17	0.00	Practices	1.86	0.43, 3.28	1778.84	0.01
	3–5	-6.03	-10.69, -1.37	0.01	Individuals	8.47	7.71, 9.23		
	6 or more	-1.23	-6.12, 3.66	0.62					

Number of Practice Nurses	Constant (1 or 2)	48.70	45.60, 51.80		Practices	2.48	0.80, 4.17	1784.79	0.20
	3–4	1.74	-2.07, 5.54	0.37	Individuals	8.50	7.73, 9.27		
	5 or more	6.43	-0.07, 12.93	0.05					

Number of Admin staff	Constant (1–5)	53.47	50.12, 56.83		Practices	2.52	1.01, 4.03	1783.59	0.11
	6–10	-5.13	-10.00, -0.27	0.04	Individuals	8.47	7.71, 9.24		
	>10	-3.86	-8.01, -0.27	0.07					

Number of Staff	Constant (1–20)	51.24	48.45, 54.03		Practices	2.91	1.24, 4.58	1787.00	0.31
	21–30	-2.58	-7.54, 2.39	0.31	Individuals	8.49	7.72, 9.26		
	>30	-1.13	-5.44, 3.19	0.61					

Practice meeting	Constant (partners only)	47.62	42.51, 52.74		Practices	2.81	1.20, 4.43	1786.06	0.37
	Partners+ Admin	6.47	-2.57, 15.51	0.16	Individuals	8.48	7.72, 9.25		
	Partners+admin +clinical	2.85	-2.67, 8.36	0.31					

CDM clinic	Constant (general clinic)	47.62	42.51, 52.74		Practices	2.92	1.18, 4.65	1787.42	0.74
	Step ladder cdm clinic	1.75	-3.16, 6.66	0.48	Individuals	8.50	7.73, 9.27		
	General+specific condition	1.40	-2.94, 5.75	0.53					

† When a predictor variable is included in the model, this p-value is generated by calculating the change in -2 log-likelihood from that of the baseline model and comparing it with the percentage points of the appropriate chi-squared distribution.

σ = standard deviation of TCI scores (either between practices or between individuals within practices as specified)

‡ In the case of a categorical variable the constant term corresponds to the estimated mean TCI score for respondents in the reference category (which is given in brackets).

When all of the above factors were included in a single multiple regression model (Table 8) the factors that were significantly associated with total TCI score were gender, tenure and the number of GPs in the practice. With all the other variables in the model the effect of professional group was no longer significant at the 5% level. In practice the variables gender, tenure and professional group were highly confounded. On average GPs tended to have the longest tenure and most of the male respondents in the sample were GPs. The effect of gender on TCI has been reduced from 6.6 in the simple regression model to 3.7 in the multiple regression model. In the simple regression model the effect of gender corresponded to the difference between male respondents and female respondents; in the multiple regression model the effect of gender corresponds mainly to the difference between male and female GPs.

**Table 8:** Multiple regression: effect of each factor on TCI total score.

Predictor (reference category)	**Test of predictor**‡			Fixed components				Random components		
	
	δ	d.f.	P	Parameter	Estimate	95% CI	p	Parameter	σ	95% CI
Gender	4.34	1	0.036	constant	54.49	49.33, 59.65	<0.001	Practices	1.29	0.02, 2.57
							
				male	3.66	0.23, 7.08	0.04	Individuals	7.93	7.22, 8.64
			
Tenure	3.85	1	0.049	tenure/year	0.13	0.00, 0.26	0.05			
			
Professional groups (GP)	7.57	4	0.109	nurse	-5.36	-9.31, -1.40	0.01			
				admin	-3.42	-6.75, -0.08	0.04			
				com nurse	-3.21	-7.56, 1.16	0.15			
				Other	-1.60	-5.48, 2.28	0.41			
			
Practice list size (≤ 3500)	5.20	2	0.074	3501–7000	-4.07	-7.43, -0.72	0.02			
				>7000	-3.15	-6.84, 0.54	0.09			
			
Number of GPs (1 or 2)	7.28	2	0.026	3–5	-2.53	-7.18, 2.13	0.29†			
				6 or more	1.79	-3.24, 6.82	0.49†			

‡ The relative importance of each predictor is assessed by comparing the change in -2 log-likelihood that occurs when a particular predictor is omitted from the final model with the percentage points of a chi-squared distribution with degrees of freedom given in the column titled d.f.

σ = standard deviation of TCI scores (either between practices or between individuals within practices as specified).

† These estimates suggest that the difference between the smallest practice and the medium size practices and the difference between the smallest practices and the largest practices are not statistically significant; however the difference between medium and large practices is 4.32 with 95% CI (1.37, 7.28).

Similarly the estimated differences between GPs and the other professional groups based on the multiple regression analysis (differences ranged from 1.6 to 5.4) were smaller in those obtained when professional group was the only explanatory variable in the model (differences ranged from 3.3 to 7.7). These differences were primarily due to the difference between female GPs and respondents in the other professional groups. Further regression analysis suggested that a more parsimonious model would include gender and either tenure or a single dummy variable contrasting GPs with other respondents with little to chose between these non-nested models.

The number of GPs in a practice is correlated with list size. Of these two variables the multiple regression indicates that the number of GPs was probably the more important predictor of total TCI score. With this variable included the overall effect of list size was significant only at the 10% level. The effects of each of these variables were similar to those observed in the initial regression analyses. The lowest TCI scores were observed from respondents from a practice with a list size of between 3501 and 7000, and from respondents in practices with three to five GPs.

### Relationship between TCI and QOF

There was no relationship between practice mean team climate scores and QOF scores (Table 5). Correlations between practice mean team climate scores and QOF clinical domains are shown in Additional File 1.

## Discussion

This study explores (1) factors correlated with team climate in general practice, and (2) the relationship between team climate and quality of care in general practice. We used the short version of TCI to measure team climate, and QOF scores as our quality outcome. We found that being male, a GP, of longer tenure in a practice and practice size (list size and number of GPs) were all significantly correlated with team climate scores. However, we found no relationship between TCI scores and QOF scores. This is consistent with our earlier review of literature which found limited evidence supporting a direct relationship between team climate and quality of care in primary care (Goh T, Eccles M: Team climate and quality of care in primary health care: a review of studies using Team Climate Inventory in the United Kingdom, Submitted) [38].

General practitioners reported higher team scores compared to "other professional roles" within general practice. This agrees with previous studies that highlighted professional differences in ownership, disciplines' philosophies and training. The status of team members can affect participation and decision making hence the team climate [15,39,40]. Contractual changes in primary care could also have an impact on the team working between staff within and beyond the usual boundary of 'the practice'. Staff who had worked longer in a practice had slightly higher team climate scores. It has been reported that teams with higher stability were found to be more effective [9,15,41]. However, it is not clear if there is an optimal duration after which an individual integrates into a team. We do not have a ready explanation for why male respondents had higher team scores compared to female

As in previous studies [29,39,40] our findings suggest smaller practices had higher team climate. However, it is uncertain whether smaller practices with higher team climate in our sample actually provide better quality of care compare to medium size practices. The larger practices in our sample were more likely to be training practices. Historically, these larger practices had also been "fund-holding" practices and were thought to be more innovative. In contrast to previous studies [15] we did not find higher team climate for practices which had more meetings.

While two previous studies have suggested an association between team climate and team effectiveness and also quality of care [29,31], we were unable to find significant relationship between team climate and QOF scores, a similar finding to studies in the UK [32] and the Netherlands [38]. The TCI was originally developed to measure team innovation, but has subsequently been used by others to predict outcomes other than innovation including intention to leave, satisfaction, sick leave, and team effectiveness [42-45]. As argued by Bosch et al [38], the type of outcome measure could affect the search of an association with quality of care. In addition there was not a large amount of variation in our measure of quality (QOF scores). This raise the possibility that whilst it could be an appropriate measure, there was insufficient variation for it to discriminate in the current study.

### Limitations

There are some limitations to this study. We used convenience sample of 14 practices which limits the generalisability of our findings. The practices were within a single PCT within one geographical area and had similar population characteristics and arrangements of community and secondary services. Because of the exploratory nature of this work we were only able to recruit a fairly small number of practices. With only 14 practices we had adequate power (80%) to detect only large associations (equivalent to an effect size of 0.65) between the TCI and our measures of quality. This section of the results where we have analysed summary statistics for each practice should be treated with caution as we may have failed to detect small but potentially important associations.

We analysed the association between team climate and QOF using data from 2006/07; whilst it was the most recent available data it predated the survey. There are criticisms of using QOF as a measure of quality of care because of its ceiling effects (as noted earlier), and whether it is an appropriate measure of quality of care as it is also a tool for performance payment. However, it is the only data publicly available in the UK general practice.

The TCI has the major advantage of being a validated instrument that can be, and has been, used in an "off the shelf" way to explore attributes of care in general practice. However, across a number of studies [31,32,38] it has not been found to have a consistent relationship with measures of quality. Given the measure's purpose this is perhaps surprising as it would be reasonable to assume that high scoring on at least three of the four subscales (vision, task orientation, support for innovation) might make the delivery of high quality care more likely. The previous studies have been compromised by study size, included individuals or team size (Goh T, Eccles M: Team climate and quality of care in primary health care: a review of studies using Team Climate Inventory in the United Kingdom, Submitted). The role of the TCI in assessing quality of care, in the UK NHS at least, should be evaluated in large study of large teams. Future study should also explore the use of other instruments compare to TCI and measurement of changes in quality of care.

## Conclusion

We found no relationship between team climate and quality of care in general practice. Gender, tenure, and number of GPs in the practice were found to be significantly correlated with team climate scores. Practices with smaller list size and number of GPs appeared to have higher team climate. Further larger studies with more complex design and using different instruments to measure dynamic processes are needed to explore the relationship between team functioning and quality of care. A number of methodological challenges need to be answered.

## Competing interests

The authors declare that they have no competing interests.

## Authors' contributions

TG designed the study, performed the data collection and data analyses under the supervision of MPE, and NS on data analyses. All authors contributed to interpreting the data. TG wrote the first draft and all authors contributed to the subsequent drafts. All authors read and approved the final manuscript.

## Pre-publication history

The pre-publication history for this paper can be accessed here:

http://www.biomedcentral.com/1472-6963/9/138/prepub

## Supplementary Material

Additional file 1 **Correlation between mean practice TCI scores and individual clinical domains**. The data provided represent the statistical analysis of the correlations between practice mean team climate scores and QOF clinical domains. Click here for file
