# Supplementary material for: Factors predicting team climate, and its relationship with quality of care in general practice
Source: BMC Health Serv Res. 2009 Aug 4;9:138. doi: 10.1186/1472-6963-9-138 (PMC3224748; doi:10.1186/1472-6963-9-138)
Supplement: Additional file 1 — Correlation between mean practice TCI scores and individual clinical domains. The data provided represent the statistical analysis of the correlations between practice mean team climate scores and QOF clinical domains. [file 1472-6963-9-138-S1.doc]

**Additional files**

**Additional file 1**

**Correlation between mean practice TCI scores and individual clinical domains.**

|  | **TCI** | **Vision** | **Participatory safety** | **Task Orientation** | **Support for Innovation** |
| --- | --- | --- | --- | --- | --- |
| **Clinical domain** |  |  |  |  |  |
| Coronary Heart Disease | 0.3011 | 0.3580 | 0.1121 | 0.3331 | 0.3812 |
| Stroke | -0.0602 | -0.0576 | 0.0136 | -0.0494 | -0.1665 |
| Hypertension | 0.2760 | 0.1966 | 0.1546 | 0.2889 | 0.4503 |
| Diabetes Mellitus | 0.0934 | -0.0060 | 0.0764 | 0.0637 | 0.2432 |
| COPD | 0.3190 | 0.3178 | 0.1402 | 0.3072 | 0.4980 |
| Epilepsy | -0.2004 | -0.1915 | -0.1429 | -0.2241 | -0.2059 |
| Cancer | -0.1791 | -0.3421 | -0.1149 | -0.1164 | -0.0887 |
| Mental Health | -0.3876 | -0.4614 | -0.3752 | -0.3424 | -0.2410 |
| Asthma | -0.0900 | -0.2491 | -0.1329 | -0.0008 | 0.0972 |
| Heart Failure | -0.0252 | -0.1481 | -0.0314 | 0.0110 | 0.1014 |
| Palliative care | -0.0455 | -0.3239 | 0.1082 | -0.0303 | 0.0617 |
| Dementia | -0.2291 | -0.3939 | -0.2340 | -0.1139 | -0.0756 |
| Depression | -0.0073 | -0.0301 | -0.1529 | 0.0536 | 0.1723 |
| Chronic Kidney Disease | 0.3422 | 0.3621 | 0.3060 | 0.3410 | 0.2625 |
| Atrial Fibrillation | 0.2743 | 0.1780 | 0.1264 | 0.2949 | 0.5007 |
| **Organisational domain** |  |  |  |  |  |
| Patient record | -0.1101 | 0.0942 | -0.2400 | -0.1397 | -0.0960 |
| Communication with patient | 0.2205 | 0.3711 | 0.1625 | 0.2451 | 0.0245 |
| Education and training | -0.1636 | -0.2397 | -0.0395 | -0.1576 | -0.2101 |
| Management | 0.2326 | 0.4346 | 0.1262 | 0.2470 | 0.0538 |
| Medicines management | -0.3205 | -0.1727 | -0.2821 | -0.4054 | -0.3589 |

Number of practice N=14

* if p<0.05; TCI=mean practice total TCI score
